# Urine Peptidome Analysis Identifies Common and Stage-Specific Markers in Early Versus Advanced CKD

**DOI:** 10.3390/proteomes11030025

**Published:** 2023-08-23

**Authors:** Sam Hobson, Emmanouil Mavrogeorgis, Tianlin He, Justyna Siwy, Thomas Ebert, Karolina Kublickiene, Peter Stenvinkel, Harald Mischak

**Affiliations:** 1 Division of Renal Medicine, Department of Clinical Science, Intervention and Technology, Karolinska Institutet, 141 52 Stockholm, Sweden; sam.hobson@ki.se (S.H.); thomas.ebert@ki.se (T.E.); karolina.kublickiene@ki.se (K.K.); peter.stenvinkel@ki.se (P.S.); 2 Mosaiques Diagnostics GmbH, 30659 Hannover, Germany; mavrogeorgis@mosaiques.de (E.M.); tinaho_ok@hotmail.com (T.H.); siwy@mosaiques-diagnostics.com (J.S.); 3 Institute for Molecular Cardiovascular Research (IMCAR), RWTH Aachen University Hospital, 52074 Aachen, Germany; 4 Medical Department III—Endocrinology, Nephrology, Rheumatology, University of Leipzig Medical Center, 04103 Leipzig, Germany

**Keywords:** CE-MS, CKD, eGFR, urine, peptides, progression

## Abstract

Given the pathophysiological continuum of chronic kidney disease (CKD), different molecular determinants affecting progression may be associated with distinct disease phases; thus, identification of these players are crucial for guiding therapeutic decisions, ideally in a non-invasive, repeatable setting. Analyzing the urinary peptidome has been proven an efficient method for biomarker determination in CKD, among other diseases. In this work, after applying several selection criteria, urine samples from 317 early (stage 2) and advanced (stage 3b–5) CKD patients were analyzed using capillary electrophoresis coupled to mass spectrometry (CE-MS). The entire two groups were initially compared to highlight the respective pathophysiology between initial and late disease phases. Subsequently, slow and fast progressors were compared within each group in an attempt to distinguish phase-specific disease progression molecules. The early vs. late-stage CKD comparison revealed 929 significantly different peptides, most of which were downregulated and 268 with collagen origins. When comparing slow vs. fast progressors in early stage CKD, 42 peptides were significantly altered, 30 of which were collagen peptide fragments. This association suggests the development of structural changes may be reversible at an early stage. The study confirms previous findings, based on its multivariable-matched progression groups derived from a large initial cohort. However, only four peptide fragments differed between slow vs. fast progressors in late-stage CKD, indicating different pathogenic processes occur in fast and slow progressors in different stages of CKD. The defined peptides associated with CKD progression at early stage might potentially constitute a non-invasive approach to improve patient management by guiding (personalized) intervention.

## 1. Introduction

Chronic kidney disease (CKD) is defined as persistent (over 3 months) structural or functional aberrations to renal tissue, detrimental for health [1]. Structural abnormalities include structures, such as polycystic kidneys, while abnormal kidney function is expressed as glomerular filtration rate < 60 mL/min/1.73 m^2^, albuminuria ≥ 30 mg/24 h or ACR ≥ 30 mg/g. CKD can be classified into G1 (≥90), G2 (60–89), G3a (45–59), G3b (30–44), G4 (15–29), G5 (<15) stages based on estimated glomerular filtration rate (eGFR), with G5 indicating kidney failure. At this stage, dialysis or transplantation are required to compensate for the loss of kidney function. CKD affects about 1 in 10 people worldwide [2] and is a major contributor to global mortality [3], with 50% of all CKD-related deaths in advanced CKD patients attributed to cardiovascular disease [4,5]. As renal function declines, uremic toxins, hyperphosphatemia, and other non-traditional CKD-specific risk factors promote persistent low-grade inflammation and a premature ageing phenotype, i.e., ‘inflammaging’ [6], further driving systemic detrimental outcomes in the body [7]. As a result, pathological processes such as vascular calcification, endothelial dysfunction and fibrosis commence take place [8]. Since the unmet clinical need of renal replacement therapy may result in premature death for millions of patients every year [9] improving our understanding of molecular players in different stages of kidney disease to slow CKD progression is crucial. Capillary electrophoresis coupled to mass spectrometry (CE-MS) is an ideal clinical application for highlighting the clinical potential of peptides and small proteins as well as determining molecular pathways involved in disease molecular pathophysiology [10,11]. Urine is highly advantageous along these lines due to its rich source of peptides/proteins and non-invasive accessibility that can be utilized in disease monitoring. In that context, over several years, the Human Urinary Proteome Database has been established, consisting of urinary peptide data of over 85,000 participants, healthy or diseased with CKD of varying stages, among other diseases. Urinary peptide data appear promising in CKD, such as in the early prediction of diabetic kidney disease (DKD) [12]. It is apparent that different pathogenic processes are initiated as CKD progresses. A particular therapeutic intervention to treat one stage can be deleterious for another, thus personalized medicine appears appropriate to target molecular players driving progression at that point in time [13]. Along these lines, exploring progression through the urinary peptidome perspective appears appropriate since the approach is applied completely non-invasively, allowing for development of panels that might distinguish progressors from non-progressors as performed by Rudnicki and colleagues [14] in the context of IgA nephropathy (IgAN). Such a tool might be of complementary importance in disease monitoring. To shed light on the different players in early and advanced CKD, data entries from almost 4000 participants of various CKD etiologies, without hyperfiltration (eGFR < 90 mL/min/1.73 m^2^) were obtained. After applying several inclusion criteria, the association of baseline urinary peptide data with eGFR progression was investigated within two groups of early and advanced CKD stages matched for relevant clinical variables, namely age, sex, body mass index (BMI), mean arterial pressure (MAP), and presence of diabetes. Using both functional gene enrichment analysis for genes coding for significant peptides and protease prediction analyses for the identification of the enzymes involved in peptide generation, pathways, and processes relevant to early and advanced CKD were proposed.

## 2. Materials and Methods

### 2.1. Initial Patient Population

**Figure 1:**
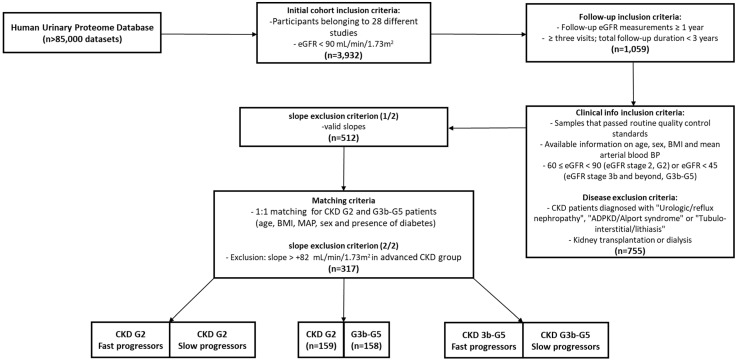
Study design. Initially, urinary peptide data for almost 4000 patients were collected from the Human Urine Proteome Database. After removing participants with eGFR ≥ 90 mL/min/1.73 m^2^ (to prevent the noise of hyperfiltration), further follow-up criteria were applied, based on both duration (at least a year of follow-up time) and number of visits (at least three visits for eGFR measurements if follow-up time < 3 years). Participants were subsequently separated into two groups based on their eGFR, namely early (G2) and advanced (G3b–5) stage CKD group. After the progression of each individual per year was calculated in terms of eGFR slopes, the individuals were matched for age, BMI, MAP, sex, and presence of diabetes, to account for potential confounding variables. Further, differential peptide abundance comparisons were performed between the groups (all individuals) as well as within groups (progressors vs. non-progressors), peptides significant after adjustment were used as an input for bioinformatics analyses.

Data entries from 3932 participants were acquired from the Human Urinary Proteome Database, using eGFR < 90 mL/min/1.73 m^2^ as a criterion to avoid cases of hyperfiltration. This database is based on more than 85,000 urinary peptide datasets analyzed through CE-MS that are highly comparable, with no detectable batch effects processed and normalized as described elsewhere [10,11]. Subsequently, 1059 baseline patient entries with follow-up eGFR measurements of at least one year and a minimum of three visits, when the total follow-up duration was less than 3 years, were obtained. Samples used in the current paper were analyzed within a maximum of 90 days of the baseline visit. After only considering samples that passed the routine quality control standards and with available information on age, sex, BMI, and MAP, as well as 60 ≤ eGFR < 90 (G2) or eGFR < 45 (G3b and beyond, G3b–G5), without “Urologic/reflux nephropathy”, “ADPKD/Alport syndrome” or “Tubulo-interstitial/lithiasis”, 755 adult patients remained for further analysis [14,15,16,17,18,19,20,21,22]. At the time of urine sampling, no patients underwent kidney transplantation or dialysis. All data are fully anonymized; thus, the current study is in agreement with the Declaration of Helsinki. Ethical review and approval were waived for this study by the ethics committee of the Hannover Medical School, Germany (no. 3116-2016), due to all data being fully anonymized. GFR estimation was based on Chronic Kidney Disease Epidemiology Collaboration (CKD-EPI) equation [23]. The MAP was calculated based on the formula: MAP = (2 × diastolic blood pressure + systolic blood pressure)/3. The study design is depicted in Figure 1.

### 2.2. CKD Progression

For the remaining 755 patients, the slopes of the linear regression models, based on the formula ‘eGFR change from baseline (%) ~ years of follow-up’, were calculated as an indication of CKD progression. Thus, based on these variables, valid slopes and their respective *p*-values, slopes could be calculated for 512 patients.

### 2.3. Matching

The remaining 512 patients were matched for age, BMI, MAP, sex, and presence of diabetes, leading to a total cohort of 318 patients forming equally sized G2 and G3b–G5 groups. A slope > +82 mL/min/1.73 m^2^ was observed for an advanced CKD patient and thus, was removed from further analysis as this patient was not deemed credible without disturbing the non-significant differences of the matched variables between the two groups. Almost all individuals had diabetes (*n* = 314), while only one patient had coronary artery disease. A total of 161 patients had CKD, of which 159 had DKD and 2 with IgAN. One patient with coronary artery disease had neither diabetes nor CKD. Clinical information is described in Table 1.

### 2.4. Peptide Differential Abundance Analyses

For the 317 matched subjects, a comparison between fast and slow progressors was performed within each group. Fast or slow progression was defined by using the following approach. Initially, each (early or advanced) CKD group was divided into tertiles based on the participants’ slopes. Participants belonging to the tertile with the most negative slopes were defined as “fast” progressors and similarly the individuals in tertiles with the positive slopes as “slow” progressors.

### 2.5. Processing of Peptides/CE-MS Analysis

Details of urine sample preparation and urinary peptidomic analysis are described elsewhere [11,12,13]. In brief, urinary analysis was performed with a P/ACE MDQ CE (Beckman Coulter, Fullerton, CA, USA) coupled to a micro-TOF-MS (Bruker Daltonic, Bremen, Germany). The Raw MS data were evaluated using the proprietary MosaFinder software (version 1.4) by applying a probabilistic clustering algorithm on isotopic distributions and conjugated mass for charge state determination. The capillary electrophoresis migration time was normalized based on the reference signal from internal peptide standards or calibrators (peptides from housekeeping proteins) using local regression. Peptide’s abundance analysis was semi-quantitatively performed in reference to 29 internal standards generally insensitive to disease. The use of internal standards allows to account for sample concentration variation factors, such as fluid intake [24]. The final result is a peak list, characterizing each protein and peptide by its molecular mass [Da] and normalized CE-migration time [min]. Normalized signal intensity is used as a measure for relative abundance. Although the MosaFinder software approach cannot be used for detecting novel compounds, mapping the identified molecules in a well-defined dataspace of 21,559 features is appropriate for biomarker analysis. Sequencing of the CE-MS detected endogenous peptide was obtained by matching of the obtained ion packs with the peptide sequences obtained by liquid chromatography-mass spectrometry analysis (LC-MS/MS). Matching was performed based on the correlation of mass between the two instruments. For further validation of obtained peptide identifications, the correlation between peptide charge at the working pH of 2 and CE-migration time was utilized to minimize false-positive identification rates [25]. The amino acid sequences were obtained by performing MS/MS analysis using an Ultimate 3000 nano-flow system (Dionex/LC Packings, Sunnyvale, CA, USA) or a P/ACE MDQ CE system (Beckman Coulter, Fullerton, CA, USA), both connected to an LTQ Orbitrap hybrid MS (Thermo Fisher Scientific, Bremen, Germany) equipped with a nano-electrospray ion source. The MS is operated in data-dependent mode to automatically switch between MS and MS/MS acquisition. Survey full-scan MS spectra (from *m*/*z* 300–2000) were acquired in the Orbitrap. Ions were sequentially isolated for fragmentation. Data files were searched against the UniProt human nonredundant database using Proteome Discoverer 2.4 and the SEQUEST search engine without enzyme specificity (activation type: HCD; precursor mass tolerance: 5 ppm; fragment mass tolerance: 0.05 Da). No fixed modifications were selected, oxidation of methionine and proline were selected as variable modifications.

### 2.6. Statistical Analysis

The results and findings of the current paper were calculated using R programming (R version 4.2.2, R Foundation for Statistical Computing, Vienna, Austria) [26]. Matching between participants of eGFR stages G2 and G3b–G5, based on age, BMI, MAP, sex, and presence of diabetes at 1:1 ratio, was performed using the MatchIt R package (version 4.5.2) through the ‘nearest neighbor’ method using logistic regression to estimate the distance-measure [27]. The algorithm was supported towards achieving more comparable variable distributions by removing participants before each matching step, with the goal that variables between the two groups did not significantly differ. The latter was based on the Mann–Whitney U test for numeric variables and Chi-square (or Fisher’s exact test for small-sized groups) test for categorical variables using stats R package functions wilcox.test (exact = FALSE) and chisq.test (or the fisher.test), respectively. The Mann–Whitney U test, for progression comparison, was based on the col_wilcoxon_twosample (exact = FALSE) function of the matrixTests R package (version 0.2.2) [28]. Peptide fragments with a *p*-value < 0.05 were considered significant, after adjustment for multiple testing (using the p.adjust (method = “BH”) function of the stats package) using the Benjamini–Hochberg method. Peptides for which sequence information was available (*n* = 5071) and present in at least 30% of the samples in the entire dataset (*n* = 1205), were analyzed.

### 2.7. Protease Analysis

Proteasix (http://www.proteasix.org, accessed on 1 March 2023), an open-source tool, was used for the protease prediction analysis [29]. The generated list of proteases was “observed” proteases, where the protease/cleavage association site was collected from literature. To improve the reliability of the proteolytic data, only “observed” proteases were analyzed. Parental proteins for all significant peptide fragments in each cohort were inputted.

### 2.8. Pathway Analysis

Enrich (http://maayanlab.cloud/Enrichr, accessed on 1 March 2023) was implemented for functional gene enrichment analysis to investigate associations in respect to Kyoto Encyclopedia of Genes and Genomes (KEGG) [30,31,32] pathways, Gene Ontology (GO) biological pathways, and GO molecular pathways to highlight the pathophysiology of the disease mechanism in terms of progression within the early CKD group.

## 3. Results

### 3.1. Cohort Determination and Baseline Characteristics

For 512 participants with follow-up information that passed the filtering criteria, valid slopes and p-values were calculated. These patients were used as a basis for the formation of two groups based on eGFR, namely G2 and G3b–G5, representing early and advanced CKD stages, respectively. The two groups were matched for relevant clinical parameters, thus eliminating the confounding potential of these established risk factors, finally leading to 318 participants, of which 317 were considered for further analyses. The study design is illustrated in Figure 1. Clinical patient data are summarized in Table 1.

### 3.2. Comparison between Early and Advanced CKD Stages

First, to better understand relevant pathophysiological processes, non-parametric Mann–Whitney U test was performed to compare differences in the baseline urinary peptidome between the G2 and G3b–G5 groups. A total of 929 urinary peptide fragments were significantly different between early and advanced CKD patients. The top 20 significant peptide fragments, with the highest or lowest fold change between strata, are presented in Table 2. Eleven different peptide fragments represent the top 20 peptides increased in advanced CKD patients. This includes three collagen subtypes, of which collagen alpha-1(I) (COL1A1) had the highest fold change (fold change: 339.2; *p* < 0.001). Three apolipoprotein A (APOA) subtypes were also present, with APOA4 representing the highest fold change (fold change: 179.2; *p* < 0.001); four alpha-1-antitrypsin (SERPINA1) fragments were also highly expressed in this group. The remaining peptides were beta-2-microglobulin, hemoglobin subunit beta, transthyretin, and mucin-19 fragments, of which the latter had the second highest fold change (fold change: 223.5; *p* < 0.001). On the contrary, the top 20 significant peptide fragments with the lowest fold change between advanced and early CKD patients are mainly COL and CD99 antigen (CD99), consisting of 12 and 3 fragments, respectively. COL21A1 was the peptide fragment with the lowest fold difference between strata (fold change: 0.0196; *p* < 0.001). The remaining peptides include semaphorin-7A, polymeric immunoglobulin receptor, calcium-dependent secretion activator 1, uromodulin, and complement C4-A.

### 3.3. Comparison Based on Progression within Early and Advanced CKD Stages

To investigate potential differences in progression between the G2 and G3b–G5 groups, comparisons between fast and slow progressors within the G2 (early CKD) and G3b–G5 groups (advanced CKD) were performed. In early CKD, a total of 42 peptides significantly differed between slow and fast progressors. Looking at the most significantly upregulated 10 peptides (Table 3), 9 were collagen (COL) fragments, with COL5A2 representing the peptide with the highest fold change (fold change: 6.6; *p* < 0.05); basement membrane-specific heparan sulfate proteoglycan core protein was also present. In a similar fashion, 7/10 peptide fragments downregulated in fast vs. slow progressors were COL fragments (lowest fold change COL9A3; fold change: 0.212; *p* < 0.05; Table 3). The remaining peptides were sodium/potassium-transporting ATPase subunit gamma, CD99 antigen, and POTE ankyrin domain family member F. However, only four peptide fragments were significantly different between fast and slow progressors in advanced CKD (Table 4). Apolipoprotein A-I demonstrated the highest fold change (fold change: 8.449; *p* < 0.05), with alpha-2-HS-glycoprotein, fibrinogen alpha chain, and COL1A1 following (note: COL1A1 was downregulated).

### 3.4. Protease Analysis between Different CKD Stages and Progressor Types

**Figure 2:**
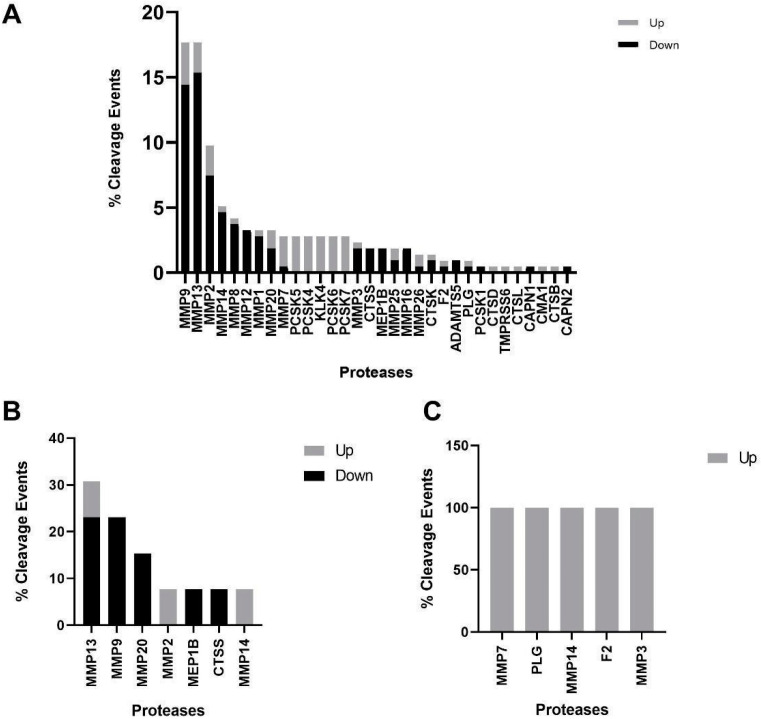
In silico predicted proteases. All significant peptides were imputed for the respective cohorts. Proteases predicted to cleave parental proteins for either upregulated or downregulated parental proteins are also noted. Data expressed as % of total cleavage events. (**A**) Early vs. late-stage CKD patients. (**B**) Fast vs. slow progressors in early CKD. (**C**) Fast vs. slow progressors in advanced CKD.

Next, we analyzed proteases known to cleave significant peptide fragments in the different cohorts. As depicted in Figure 2, when comparing early and advanced stage CKD patients, 32 proteases were responsible for 215 predicted cleavage events, of which 144 and 71 corresponded to downregulated and upregulated peptides, respectively. The nine proteases with the highest number of cleavage events were matrix metalloproteinases (MMPs), responsible for 138 (64%) cleavage events, primarily for downregulated peptides. Interestingly, PCSK5, PCSK4, KLK4, PCSK6, and PCSK7 were predicted to cleave six peptides each, all of which are downregulated in advanced CKD patients. Only 13 peptide cleavage events were predicted when comparing slow and fast progressors in early CKD patients. The majority of events were in peptides downregulated in fast progressors, 11/13 (85%) of which were MMPs. Due to the low number of significant peptides in the advanced CKD cohort, only five peptide cleavage events were determined, with all proteases predicted to cleave FGA.

### 3.5. Functional Pathway Analysis between Different CKD Stages and Progressor Types

Lastly, we performed functional pathway analysis using genes that code for significant peptides in the early CKD cohort. For optimum coverage, all significant peptides were analyzed. The top three GO terms were compared between strata. We found Go Biological Processes related to extracellular matrix (ECM) organization (GO:0030198 and GO:0043062) were enriched for significantly downregulated and upregulated peptides in this cohort. GO Cellular Components terms were identical between strata; however, Molecular Function terms differed slightly between groups. While Protease Binding (GO:0002020) was highly enriched in both strata, only five enriched functions were present for upregulated peptides for Go Molecular Function terms. Platelet-Derived Growth Factor Binding was ranked number one (GO:0048407).

## 4. Discussion

It is currently unclear whether the kidney function of a patient with CKD will have a fairly stable course or a rapid decline. Nevertheless, this information is essential in the context of guiding therapeutic decisions in a personalized manner, given that a patient-oriented approach and early intervention is generally expected to maximize therapeutic results. Considering the CKD pathophysiological continuum, molecular determinants responsible for a patient’s disease progression are expected to be stage-specific. In this context, a phase-specific investigation of molecular mechanisms in terms of CKD progression is warranted. To this end, we aimed to highlight stage-specific molecular signatures associated with disease as well as disease progression. The urinary proteome was investigated since naturally occurring peptides and small proteins were analyzed. CE-MS technology that enables separation and detection of the highly complex urinary proteome/peptidome was applied. Understanding of the complex proteome or peptidome is crucial for gaining a comprehensive view of biological processes and disease mechanisms. The current study concurs with previous findings in CKD progression [33] and provides further insights given its large initial cohort, design that accounts for relevant confounders and added bioinformatics perspective.

By employing this study design, a comparison between matched early and advanced CKD patients was carried out to determine the parental proteins of potential importance in the respective disease stages. Collagen fragments accounted for the majority (648/929) of the significant urinary peptides in this analysis. This is not surprising considering the fibrotic activity of collagens as a part of the extracellular matrix and its turnover. In the context of CKD, such urinary peptide data have been abundantly observed, e.g., by Schanstra et al. (2015) [34]. Of course, fibrotic events do not occur exclusively in renal tissue in CKD; increased collagen expression has been observed in vascular remodeling ultimately leading to vascular calcification and increased stiffness, a sequalae of CKD. In addition, increased deposition of extracellular matrix proteins, driven by uremia, is often observed in cardiac tissue, with heart failure the common end-result. Thus, it must be pointed out that changes in the peptidome may reflect peptides originating from tissue other than the kidney. Examples of fragments of non-collagen origin were derived, among others, from proteins with inflammatory function, e.g., polymeric immunoglobulin receptor, alpha-1-antitrypsin, complement C4-A, and semaphorin-7A. This does not come as a surprise, since ‘inflammaging’ is a risk factor associated with CKD mortality.

Comparing slow vs. fast progressors within the early CKD stages revealed 42 significant peptides derived from 21 parental proteins, 12 of which were again, collagen molecules. Nevertheless, with regards to the non-collagen fragments, peptides were derived from CD99 antigen, fibrinogen alpha protein, and uromodulin, which were also among the most significant in the respective eGFR strata (60–90 mL/min/1.73 m^2^) progression comparisons performed by Pontillo et al. (2017) [33]. In the same study, fragments from the basement membrane-specific heparan sulfate proteoglycan core protein were also found to be most significant, but only in the 40–49 mL/min/1.73 m^2^ eGFR stratum, whereas peptides of mucin-16, plasminogen, POTE ankyrin domain family member F, sodium/potassium-transporting ATPase subunit gamma and titin, as identified in our work, were not recorded as the most significant peptides in theirs.

On the other hand, association with progression in the advanced CKD group revealed limited outputs. Peptides from apolipoprotein A-I, alpha-2-HS-glycoprotein, fibrinogen alpha chain, and collagen alpha-1(I) were statistically significant. Peptides derived from collagen alpha-1(I) were also among the most significant in the work of Pontillo et al. within the same eGFR range [33]. An association between higher serum apolipoprotein A-I protein and lower prevalence of CKD as well higher eGFR has been described in CKD patients [35]. In a recent study, urinary peptide levels of alpha-2-HS-glycoprotein demonstrated significant inverse association with eGFR and eGFR slope (%) per year in type 2 diabetic patients, indicating the association of these peptides with CKD progression [36].

After the differential abundance analyses, protease prediction and pathway analysis followed. MMPs and PCSKs were predicted to be responsible for the majority of cleavage events in all three cohorts. Indeed, both proteases have been associated with peptide cleavage in cardiorenal syndrome and CKD. MMP2 and MMP9 are established proteases that degrade the ECM in CKD, thus it comes as no surprise that these rank in the top three proteases in terms of cleavage events in our cohorts. MMP13, despite its high % of cleavage events throughout, is less reported than its MMP counterparts MMP2/9 in the context of CKD progression and has even been shown to have anti-inflammatory properties [37]. Its exact role in CKD progression merits further research. PCSKs have previously been shown by our group to be implicated in CKD patients vs. non-CKD controls [38]. Our data validate findings from this study, while also emphasizing its importance in advanced CKD vs. early CKD patients, since PCSKs were predicted to cleave peptides that were upregulated in such sub cohorts.

Significant peptides associated with progression within the early CKD stages were only considered for pathway analysis (Supplementary Figure S1). Intervention appears most promising to establish a more stable course (and thus justifiably warrants more emphasis on deciphering the disease mechanism). On the other hand, in later CKD stages, the kidneys are severely damaged (while also “pressured” to address their original purpose) resulting in peptides derived from a number of plasma proteins that are inconsistently found in urine due to the presence of proteinuria; thus only a handful of peptides end up being significantly different between progressors and non-progressors.

A major advantage of our study is the non-invasive approach harnessing the capacity of the CE-MS technique. Another key strength is the initial large sample size of both cohorts, allowing us to hone in on patients with advanced kidney disease, as well as matching patients for potential confounders. Nevertheless, at the same time, we also acknowledge shortcomings of our study design, including incomplete clinical records for some variables, e.g., proteinuria, preventing adjustment for these confounders. Lastly, the present study is of a retrospective cross-sectional design, however the multicenter design, strict inclusion/statistical criteria along with, at times, a high level of significance is expected to, in part, counteract potential bias. Considering the molecular differences associated with progression, in addition to shedding light on relevant underlying mechanisms, our study might pave the way for developing classifiers with the capacity to distinguish progressors from their non-progressor counterparts, e.g., as applied by Rudnicki and colleagues [14] in a cohort of 209 biopsy-proven IgAN patients. Utilizing this approach within a non-invasive framework might constitute a powerful complementary tool in clinical practice for disease monitoring purposes that can pragmatically support gold standard methods in the challenges of the modern healthcare system.

## 5. Conclusions

In conclusion, this study provides further insight into molecular mechanisms involved in CKD progression based on the urinary proteome. Urinary peptides associated with early and advanced disease stages as well as with progression within these two different disease phases were defined and found to be distinctly different. A number of collagen-derived peptides were significantly associated with CKD progression at the early disease stage This association suggests the development of structural changes that may be reversible at an early stage. The obtained results indicate that different pathogenic processes occur in fast and slow progressors in different stages of CKD. Thus, combining early CKD progression-associated molecular features into a model for classifying individuals into progressors or non-progressors might constitute a non-invasive approach to improve patient management by guiding (personalized) intervention.

## Figures and Tables

**Table 1:** Clinical patient information for the final cohort (*n* = 317) stratified by two matched groups (early stage, G2 and advanced stage CKD, G3b–G5). The number in each group is stated. Data are presented as mean (standard deviation) for numerical variables and as a percentage for categorical variables. eGFR: estimated glomerular filtration rate; BMI: body mass index; MAP: mean arterial pressure.

Characteristics	Early Stage CKD (G2)	Advanced Stage CKD (G3b–G5)
*n*	159	158
eGFR (mL/min/1.73 m^2^)	78.6 (8.7)	31.1 (7.4)
Age	62.8 (6.2)	63.1 (9.4)
BMI (kg/m^2^)	29.5 (4.3)	30.2 (5.4)
MAP (mmHg)	96.5 (7.6)	96.1 (8.6)
Male (%)	47.8	58.9
Diabetic (%)	98.7	99.4

**Table 2:** Comparison between early (G2) vs. advanced (G3b–5) CKD groups. The top 20 significant peptide fragments, with the highest or lowest fold change based on the differential peptide abundance analysis are listed. The first half of the table refers to upregulated peptides, while the second half to downregulated. Fold change refers to the ratio of mean relative peptide abundance of advanced CKD group to early CKD group.

Protein Symbol	Protein Name	Sequence	Fold Change	Adj. *p*-Value
**Upregulated**				
COL1A1	Collagen alpha-1(I)	RGPpGPpGKNGDDGEAGKPGRpGERGPpGP	339.2	4.14 × 10^−24^
MUC19	Mucin-19	GVTGKSGLSAGVTGKTGLSAGVTGTTGPS	223.5	2.49 × 10^−17^
APOA4	Apolipoprotein A-IV	RQKLGPHAGDVEGHLS	179.2	1.88 × 10^−26^
APOA1	Apolipoprotein A-I	LEEYTKKLNTQ	161.8	7.66 × 10^−24^
HBB	Hemoglobin subunit beta	FESFGDLSTPDAVMGNPKVKAHGKKVLG	105.4	3.33 × 10^−25^
COL1A1	Collagen alpha-1(I)	PGPAGPPGEAGKPGEQGVPGDLGAPGPSGARG	95.6	1.79 × 10^−21^
APOA2	Apolipoprotein A-II	FVELGTQPATQ	87.6	1.35 × 10^−31^
TTR	Transthyretin	LSPYSYSTTAVVTNPKE	84.6	1.56 × 10^−25^
HBB	Hemoglobin subunit beta	VHLTPEEKSAVTALWGKVNVDEV	80.8	6.30 × 10^−22^
SERPINA1	Alpha-1-antitrypsin	SEGLKLVDKFLEDVKKL	71.3	7.52 × 10^−17^
SERPINA1	Alpha-1-antitrypsin	EDPQGDAAQKTDTSHHDQDHPTFNKITPN	69.2	4.61 × 10^−20^
SERPINA1	Alpha-1-antitrypsin	MIEQNTKSPLFMGKVVNPTQK	67.8	1.36 × 10^−27^
APOA1	Apolipoprotein A-I	ALEEYTKKLNTQ	67.2	2.06 × 10^−18^
COL19A1	Collagen alpha-1(XIX)	GPEGPSGKpGINGKDGIPGAQGImGKpGDRGpKGERGDQGIP	67.0	3.40 × 10^−35^
COL3A1	Collagen alpha-1(III)	GEPGRDGVPGGPGMRGMPGSPGGPGSDGKPGPpGSQGESGRpGpP	65.4	2.54 × 10^−17^
B2M	Beta-2-microglobulin	NGERIEKVEHSDLSFSKDWS	62.7	1.96 × 10^−17^
APOA1	Apolipoprotein A-I	DEPPQSPWDRVKDL	62.7	7.95 × 10^−14^
B2M	Beta-2-microglobulin	LKNGERIEKVEHSDLSFSKDWS	61.1	4.84 × 10^−22^
SERPINA1	Alpha-1-antitrypsin	EAIPMSIPPEVKFNKP	59.4	4.72 × 10^−27^
B2M	Beta-2-microglobulin	YVSGFHPSDIEVD	58.9	3.69 × 10^−15^
**Downregulated**				
COL21A1	Collagen alpha-1(XXI)	pGYPGQpGQDGKPGYQGIAGTpGVpGSPG	0.0196	3.44 × 10^−27^
COL1A1	Collagen alpha-1(I)	PpGpAGFAGpPGADGQPGAKGEPGDAGAKGDAGPPGPAGP	0.0245	3.75 × 10^−27^
COL5A3	Collagen alpha-3(V)	IDGSpGEKGDPGDVGGPGPPGASGEPGAPGPPGKRGPS	0.0287	2.83 × 10^−18^
SEMA7A	Semaphorin-7A	FREAQHWQLLPEDGIM	0.0341	1.03 × 10^−36^
COL1A1	Collagen alpha-1(I)	GADGQpGAKGEpGDAGAKGDAGPpGPAGPAGPpGPIG	0.0364	2.69 × 10^−32^
PIGR	Polymeric immunoglobulin receptor	AVADTRDQADGSRASVDSGSSEEQGGSSRALVSTLVPLG	0.0392	2.03 × 10^−15^
COL1A1	Collagen alpha-1(I)	pPGADGQPGAKGEpGDAGAKGDAGPpGPAGPAGPPGPIG	0.0406	1.14 × 10^−25^
CADPS	Calcium-dependent secretion activator 1	GGAGAGAGVGAGGGGGSGASSGGGAGGL	0.0423	4.16 × 10^−24^
COL1A1	Collagen alpha-1(I)	TGPIGpPGPAGAPGDKGESGpSGPAGPTG	0.0426	6.30 × 10^−34^
CD99	CD99 antigen	DGVSGGEGKGGSDGGGSHRKEGEEADAPGVIPGIVGA	0.0511	1.73 × 10^−25^
CD99	CD99 antigen	DLADGVSGGEGKGGSDGGGSHRKEGEEADAPGVIPG	0.0571	4.35 × 10^−23^
COL2A1	Collagen alpha-1(II)	GpAGpPGEKGEPGDDGPSGAEGpPGPQ	0.0627	1.14 × 10^−18^
COL1A2	Collagen alpha-2(I)	GEPGSAGPQGPPGPSGEEGKRGPNGEAGSAGPPGpPGL	0.0644	7.72 × 10^−44^
COL1A1	Collagen alpha-1(I)	GADGQpGAKGEpGDAGAKGDAGPPGPAGPAGPpGPIG	0.0652	1.96× 10^−33^
COL15A1	Collagen alpha-1(XV)	VSFVTGYGGFPAYSFGPGANVGR	0.0662	2.04 × 10^−20^
UMOD	Uromodulin	IDQSRVLNLGPITR	0.0686	4.16 × 10^−18^
C4A	Complement C4-A	DELPAKDDPDAPLQPVTP	0.0688	1.11 × 10^−29^
CD99	CD99 antigen	DGGFDLSDALPDNENKKPtAIP	0.0701	4.64 × 10^−36^
COL1A1	Collagen alpha-1(I)	pPGADGQpGAKGEpGDAGAKGDAGPpGPAGP	0.0729	1.07 × 10^−22^
COL1A2	Collagen alpha-2(I)	PAGSRGDGGPpGMTGFpGAAGRTGpPGPSGISGPPGPPGPAG	0.0738	5.55 × 10^−19^

**Table 3:** Comparison between progressors and non-progressors within the early stage (G2) CKD group. The top 10 significant peptide fragments, with the highest or lowest fold change based on the differential peptide abundance analysis are listed. The first half of the table refers to upregulated peptides, while the second half to downregulated. Fold change refers to the ratio of mean relative peptide abundance of progressors to non-progressors.

Protein Symbol	Protein Name	Sequence	Fold Change	Adj. *p*-Value
**Upregulated**				
COL5A2	Collagen alpha-2(V)	GSPGTSGppGSAGpPGSpG	6.6238	2.43 × 10^−2^
HSPG2	Basement membrane-specific heparan sulfate proteoglycan core protein	LAFPGHVFSRSLPEVPETIEL	5.3553	3.57 × 10^−2^
COL5A3	Collagen alpha-3(V)	GPpGPpGFpGDPGPPG	4.5915	3.24 × 10^−2^
COL5A3	Collagen alpha-3(V)	GPpGPpGFPGDpGPpG	4.1514	1.51 × 10^−2^
COL4A1	Collagen alpha-1(IV)	GPpGFTGppGPPGPPGP	3.9258	2.43 × 10^−2^
COL1A1	Collagen alpha-1(I)	GEPGSPGENGApGQMGp	3.6261	2.43 × 10^−2^
COL11A1	Collagen alpha-1(XI)	GPpGDDGMRGEDGEIGpRGLp	3.6150	6.51 × 10^−3^
COL3A1	Collagen alpha-1(III)	AGIpGVpGAKGEDGKDGSpGEpGANG	3.2461	4.07 × 10^−2^
COL1A1	Collagen alpha-1(I)	ADGQPGAKGEPGDAGAKGDAGpPGPA	2.8850	2.43 × 10^−2^
COL3A1	Collagen alpha-1(III)	pGARGLpGpPGSNGNPGpP	2.8372	6.51 × 10^−3^
**Downregulated**				
COL9A3	Collagen alpha-3(IX)	GpAGPpGpPGPpG	0.2119	3.24 × 10^−2^
COL1A2	Collagen alpha-2(I)	TGPPGPSGISGPpGpPGPAG	0.2423	3.69 × 10^−2^
COL22A1	Collagen alpha-1(XXII)	pGVpGPPGPGGSPGLPGE	0.2741	2.43 × 10^−2^
COL3A1	Collagen alpha-1(III)	PpGENGKpG	0.3418	3.90 × 10^−2^
COL4A3	Collagen alpha-3(IV)	GPPGTpGEpGMQGEpGPP	0.3592	1.60 × 10^−2^
FXYD2	Sodium/potassium-transporting ATPase subunit gamma	TGLSMDGGGSPKGDVDP	0.3857	3.24 × 10^−2^
CD99	CD99 antigen	DGVSGGEGKGGSDGGGSHRKEGEEADAPGVIPGIVGAVV	0.3898	1.00 × 10^−2^
COL3A1	Collagen alpha-1(III)	SpGERGETGPpGPA	0.3976	2.43 × 10^−2^
POTEF	POTE ankyrin domain family member F	RVAPEEHPV	0.3984	1.51 × 10^−2^
COL3A1	Collagen alpha-1(III)	KNGETGPQGppGPTGPGGDKGDTGPpGPQG	0.4087	2.43 × 10^−2^

**Table 4:** Comparison between progressors and non-progressors within the advanced stage (G3b–5) CKD group. Significant peptide fragments based on the differential peptide abundance analysis are listed. Fold change refers to the ratio of mean relative peptide abundance of progressors to non-progressors.

Protein Symbol	Protein Name	Sequence	Fold Change	Adj. *p*-Value
APOA1	Apolipoprotein A-I	ALEEYTKKLNTQ	8.4493	1.94 × 10^−2^
AHSG	Alpha-2-HS-glycoprotein	LGSPSGEVSHPRKT	7.6154	4.51 × 10^−3^
FGA	Fibrinogen alpha chain	SGEGDFLAEGGGVR	2.1838	1.94 × 10^−2^
COL1A1	Collagen alpha-1(I)	NSGEpGApGSKGDTGAkGEpGPVG	0.4708	4.35 × 10^−2^

## Data Availability

Data will be made available upon request directed to the corresponding author. Proposals will be reviewed and approved by the investigators and collaborators based on scientific merit. After approval of a proposal, data will be shared through a secure online platform after signing the data access and confidentiality agreement.

## Supplementary Materials

The following supporting information can be downloaded at: https://www.mdpi.com/article/10.3390/proteomes11030025/s1, Table S1: Peptide differential abundance analyses, Figure S1: Functional pathway analysis with regards to progression within the early CKD group.
