# Supplementary material for: Urine Peptidome Analysis Identifies Common and Stage-Specific Markers in Early Versus Advanced CKD
Source: Proteomes. 2023 Aug 23;11(3):25. doi: 10.3390/proteomes11030025 (PMC10534506; doi:10.3390/proteomes11030025)
Supplement: Supplementary file 1 [file proteomes-11-00025-s001.zip › Hobson_Supplementary Figure S1 - Functional pathway analysis.pdf]

Early_Downregulated_Process

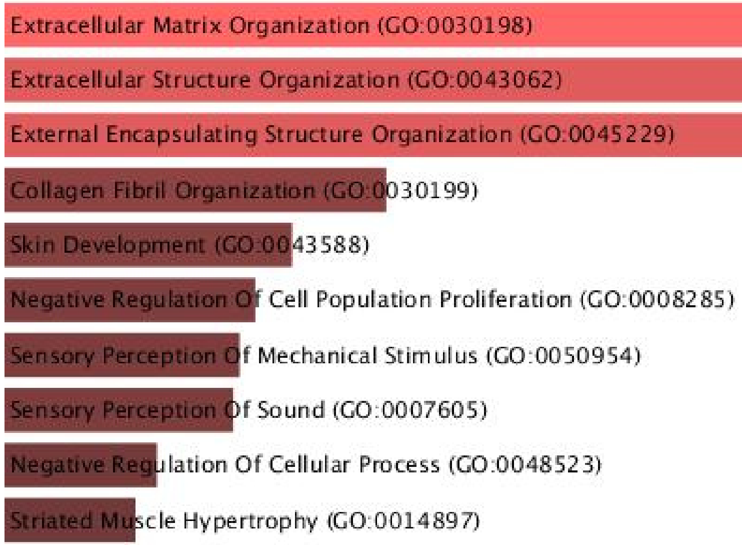

Index	Name	P-value	Adjusted p-value	Odds Ratio	Combined score
1	Extracellular Matrix Organization (GO:0030198)	1.212e-8	0.000001855	115.90	2112.67
2	Extracellular Structure Organization (GO:0043062)	1.709e-7	0.000009043	126.25	1967.31
3	External Encapsulating Structure Organization (GO:0045229)	1.773e-7	0.000009043	125.06	1944.05
4	Collagen Fibril Organization (GO:0030199)	0.0001917	0.007331	124.69	1067.29
5	Skin Development (GO:0043588)	0.0005036	0.01541	75.47	573.09
6	Negative Regulation Of Cell Population Proliferation (GO:0008285)	0.0007338	0.01759	22.36	161.35
7	Sensory Perception Of Mechanical Stimulus (GO:0050954)	0.0008609	0.01759	57.19	403.64
8	Sensory Perception Of Sound (GO:0007605)	0.0009195	0.01759	55.28	386.48
9	Negative Regulation Of Cellular Process (GO:0048523)	0.002006	0.03185	15.61	96.99
10	Striated Muscle Hypertrophy (GO:0014897)	0.002498	0.03185	555.17	3326.7

Early_Downregulated_Component

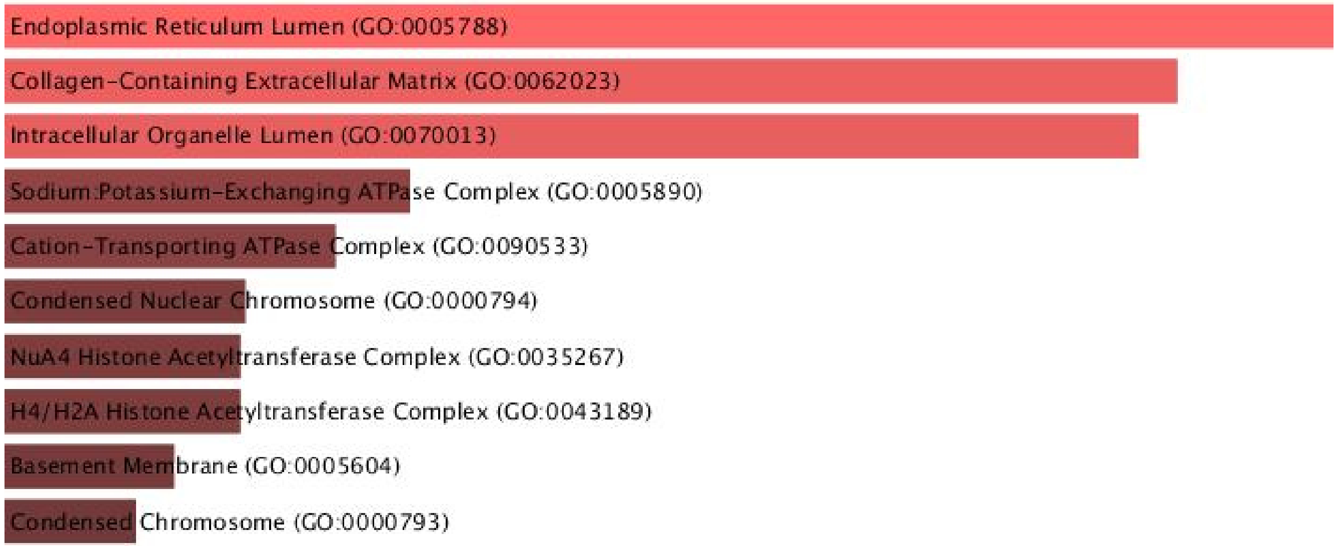

Index	Name	P-value	Adjusted p-value	Odds Ratio	Combined score
1	Endoplasmic Reticulum Lumen (GO:0005788)	0.000007815	0.0001563	46.93	551.85
2	Collagen-Containing Extracellular Matrix (GO:0062023)	0.00002287	0.0001992	35.45	378.79
3	Intracellular Organelle Lumen (GO:0070013)	0.00002989	0.0001992	22.49	234.30
4	Sodium:Potassium-Exchanging ATPase Complex (GO:0005890)	0.004492	0.02246	277.53	1500.17
5	Cation-Transporting ATPase Complex (GO:0090533)	0.007476	0.02991	158.54	776.21
6	Condensed Nuclear Chromosome (GO:0000794)	0.01392	0.03602	82.15	351.18
7	NuA4 Histone Acetyltransferase Complex (GO:0035267)	0.01441	0.03602	79.21	335.86
8	H4/H2A Histone Acetyltransferase Complex (GO:0043189)	0.01441	0.03602	79.21	335.86
9	Basement Membrane (GO:0005604)	0.02277	0.05060	49.25	186.27
10	Condensed Chromosome (GO:0000793)	0.02960	0.05913	37.53	132.12

Early_Downregulated_Function

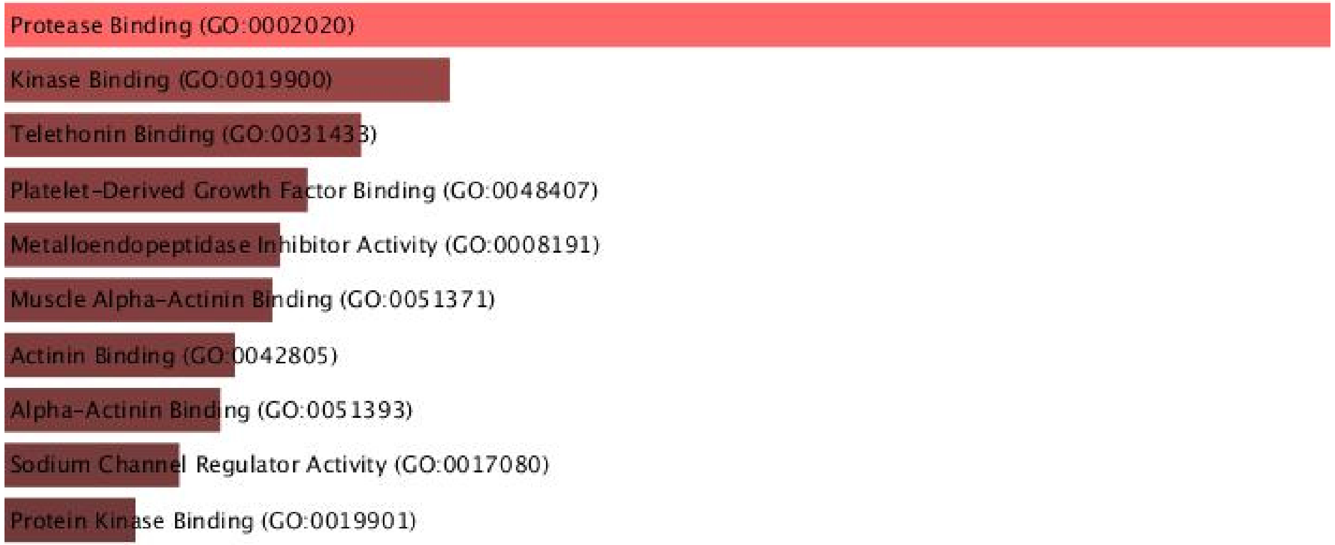

Index	Name	P-value	Adjusted p-value	Odds Ratio	Combined score
1	Protease Binding (GO:0002020)	2.966e-7	0.000005338	109.47	1645.45
2	Kinase Binding (GO:0019900)	0.001286	0.01157	18.32	121.93
3	Telethonin Binding (GO:0031433)	0.002997	0.01798	444.11	2580.41
4	Platelet-Derived Growth Factor Binding (GO:0048407)	0.004990	0.02094	246.68	1307.49
5	Metalloendopeptidase Inhibitor Activity (GO:0008191)	0.006482	0.02094	184.98	932.06
6	Muscle Alpha-Actinin Binding (GO:0051371)	0.006979	0.02094	170.74	847.70
7	Actinin Binding (GO:0042805)	0.009957	0.02560	116.79	538.34
8	Alpha-Actinin Binding (GO:0051393)	0.01144	0.02575	100.85	450.83
9	Sodium Channel Regulator Activity (GO:0017080)	0.01687	0.03375	67.20	274.29
10	Protein Kinase Binding (GO:0019901)	0.02559	0.04607	9.57	35.07

Early_Upegulated_Process

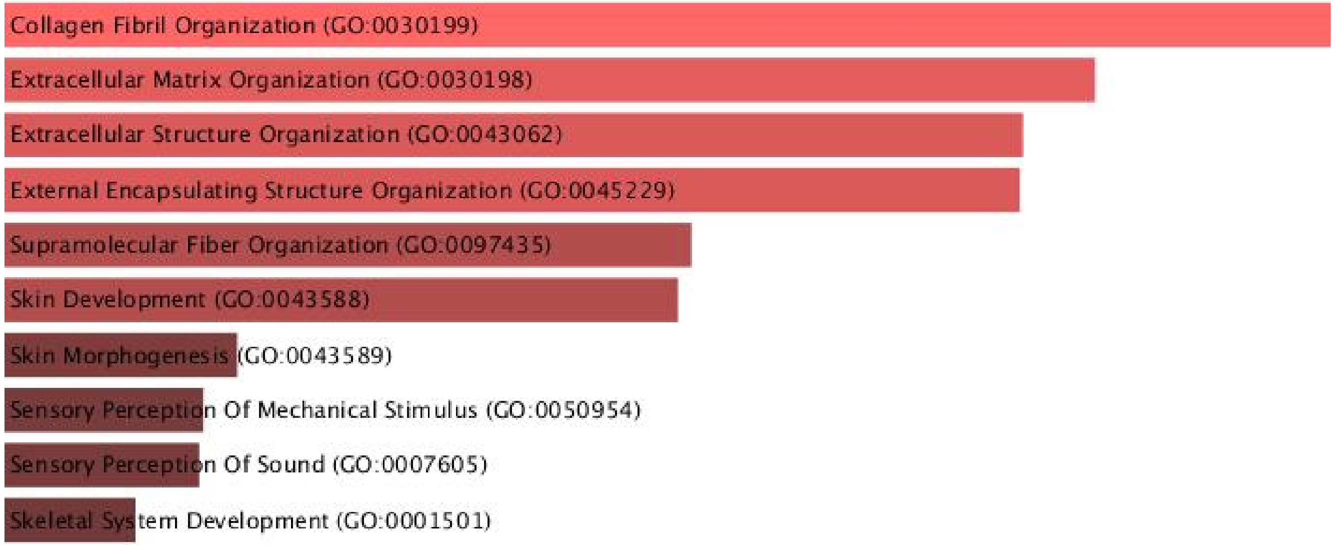

Index	Name	P-value	Adjusted p-value	Odds Ratio	Combined score
1	Collagen Fibril Organization (GO:0030199)	1.807e-16	2.204e-14	665.07	24108.49
2	Extracellular Matrix Organization (GO:0030198)	3.797e-14	2.316e-12	188.75	5832.81
3	Extracellular Structure Organization (GO:0043062)	1.961e-13	6.387e-12	227.44	6654.98
4	External Encapsulating Structure Organization (GO:0045229)	2.094e-13	6.387e-12	225.22	6575.29
5	Supramolecular Fiber Organization (GO:0097435)	3.639e-10	8.878e-9	74.30	1614.78
6	Skin Development (GO:0043588)	4.928e-10	1.002e-8	197.66	4236.02
7	Skin Morphogenesis (GO:0043589)	0.00001090	0.0001899	605.48	6918.95
8	Sensory Perception Of Mechanical Stimulus (GO:0050954)	0.00002359	0.0003532	69.42	739.68
9	Sensory Perception Of Sound (GO:0007605)	0.00002605	0.0003532	67.07	707.97
10	Skeletal System Development (GO:0001501)	0.0001097	0.001339	40.77	371.72

Early_Upregulated_Component

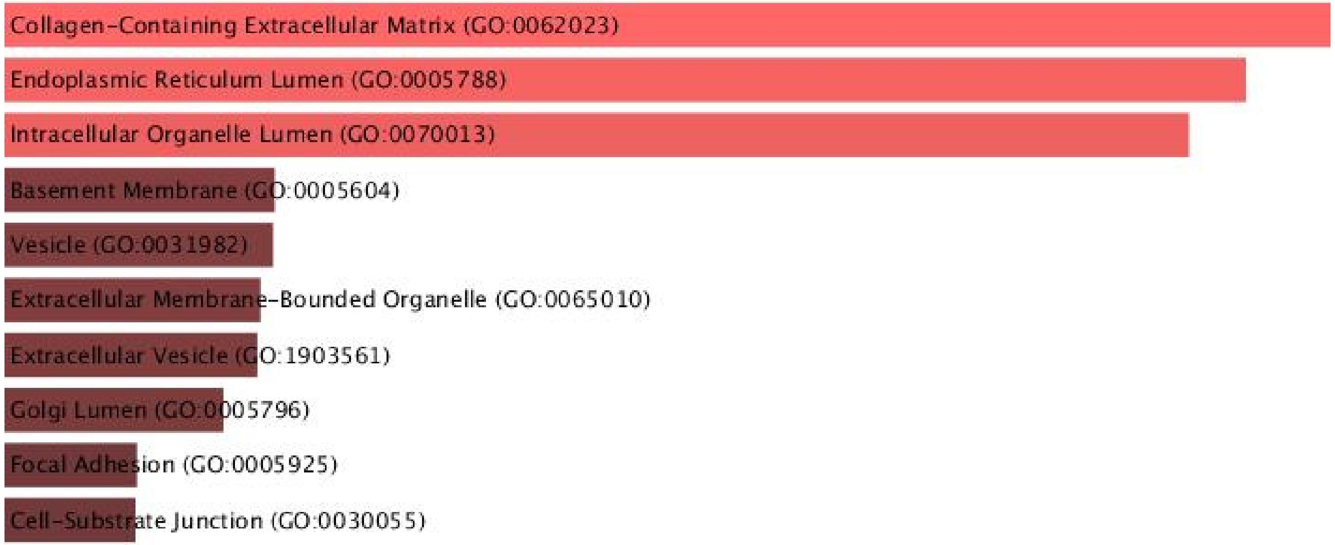

Index	Name	P-value	Adjusted p-value	Odds Ratio	Combined score
1	Collagen-Containing Extracellular Matrix (GO:0062023)	6.184e-18	9.894e-17	298.17	11814.87
2	Endoplasmic Reticulum Lumen (GO:0005788)	7.834e-17	6.268e-16	239.82	8893.73
3	Intracellular Organelle Lumen (GO:0070013)	4.381e-16	2.336e-15	272.18	9625.25
4	Basement Membrane (GO:0005604)	0.0003972	0.001344	82.41	645.35
5	Vesicle (GO:0031982)	0.0004199	0.001344	25.55	198.63
6	Extracellular Membrane-Bounded Organelle (GO:0065010)	0.0006100	0.001545	65.89	487.73
7	Extracellular Vesicle (GO:1903561)	0.0006758	0.001545	62.47	456.03
8	Golgi Lumen (GO:0005796)	0.001862	0.003725	36.90	231.95
9	Focal Adhesion (GO:0005925)	0.02530	0.04205	9.26	34.04
10	Cell-Substrate Junction (GO:0030055)	0.02628	0.04205	9.07	32.99

Early_Upregulated_Function

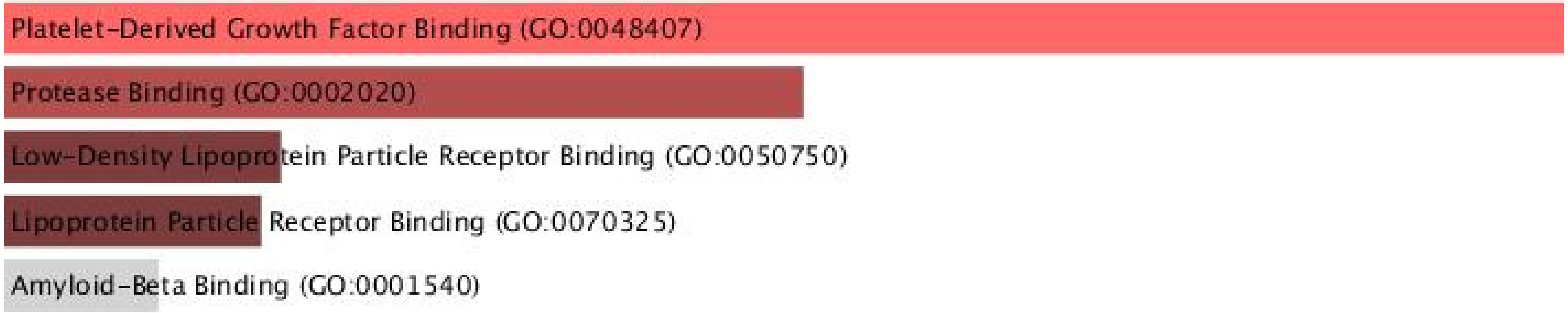

Index	Name	P-value	Adjusted p-value	Odds Ratio	Combined score
1	Platelet-Derived Growth Factor Binding (GO:0048407)	2.567e-8	1.284e-7	856.29	14966.03
2	Protease Binding (GO:0002020)	0.00006511	0.0001628	48.85	470.87
3	Low-Density Lipoprotein Particle Receptor Binding (GO:0050750)	0.01421	0.02177	79.23	337.03
4	Lipoprotein Particle Receptor Binding (GO:0070325)	0.01741	0.02177	63.98	259.14
5	Amyloid-Beta Binding (GO:0001540)	0.05016	0.05016	21.27	63.65
